# From the Cochrane Library: Skin Care Interventions in Infants for Preventing Eczema and Food Allergy

**DOI:** 10.2196/30197

**Published:** 2021-09-02

**Authors:** Jarett Anderson, Torunn Sivesind

**Affiliations:** 1 Midwestern University Glendale, AZ United States; 2 University of Colorado School of Medicine Aurora, CO United States

**Keywords:** eczema, atopic dermatitis, skin care, emollients, pediatrics, food allergy, allergy, dermatology

Eczema is a common and taxing condition, with an estimated prevalence of 10.7% among pediatric patients in the United States and a cost of US $5 billion annually [1]. Eczema has a known association with food allergies, with both conditions commonly developing during the first year of life. The cost of care and daily attention required to treat both eczema and food allergy represent significant burdens to individuals and families. Without a global standard for neonatal or infant skin care, and with few emollient studies performed in term infants, Kelleher et al’s Cochrane review [2] provides a much-needed assessment of the evidence for emollients and other interventions to prevent eczema, as well as their effects on the development of food allergy.

This systematic review assessed 33 randomized controlled trials (n=25,827), all of which studied term (>37 weeks) infants (<12 months) without a pre-existing diagnosis of eczema, food allergy, or other skin condition. Clinically relevant findings are summarized in Table 1.

**Table 1:** Clinically relevant findings.

Outcomes	Comparison	Relative risk (RR) or hazard ratio (HR) (CI)	Number of studies and participants in the pooled analysis	Quality of evidence
Development of eczema by 1-2 years	Skin care interventions vs standard care^a^	RR=1.03 (CI 0.81-1.31)	7 trials, 3075 participants	Moderate
Time needed to develop eczema	Skin care interventions vs standard care	HR=0.86 (CI 0.65-1.14)	9 trials, 3349 participants	Moderate
Development of skin infections	Skin care interventions vs standard care	RR=1.34 (CI 1.02-1.77)	6 trials^b^, 2728 participants	Moderate
IgE^c^-mediated food allergies at 1-2 years	Skin care interventions vs standard care	RR=2.53 (CI 0.99-6.47)	1 trial, 996 participants	Very low
Sensitization to food allergens at 1-2 years	Skin care interventions vs standard care	RR=0.86 (CI 0.28-2.69)	2 trials, 1055 participants	Very low

^a^Standard care is defined as no skin care or care as usual.

^b^While 2 out of the 6 studies in the pooled analysis slightly favored skin care interventions (not statistically significant), the pooled data suggested an increased risk of skin infection with emollients. The studies contributing to the pooled data varied in a number of study-related and patient-specific characteristics.

^c^IgE: immunoglobulin E.

There was limited evidence concerning the impact of skin care interventions on IgE (immunoglobulin E)-mediated food allergies; the few trials that investigated these outcomes produced broad CIs that failed to achieve statistical significance.

An important strength of this review was its inclusion of 7 studies in a meta-analysis evaluating study-specific factors (eg, type of emollient, duration) and participant covariates (eg, age, FLG [filaggrin] genotype, family history of atopy), which revealed no interplay between these factors and the intervention on eczema risk. Other potential confounders (such as bath water composition, detergent type, diet, climate and geographical factors, and dust and home allergens) were not assessed.

Further, the intervention and control groups varied widely between the included studies such that single and combination emollients of differing contents were considered together. The control (standard care) was likewise variable, depending upon national standards and cultural norms. These limitations highlight the need for additional research to improve the generalizability of the results to diverse populations.

Efforts to identify the effects of different skin care interventions on the prevention of eczema and their effects on food allergy are also warranted. There are currently a number of ongoing clinical trials assessing skin care interventions for the prevention of atopic dermatitis and food allergy; one trial recently concluded there is no evidence that the use of daily emollients reduces the risk of eczema by the age of 2 years in high-risk patients (patients with first-degree relatives with a history of eczema, asthma, or allergic rhinitis) [3].

The incidence of eczema has increased, especially since the onset of the COVID-19 pandemic. With an enhanced emphasis on frequent hand washing, hand hygiene has become an increasingly popular topic among individuals and families [4]. In recent years prior to the pandemic, an increase in the incidence of eczema in the pediatric population was reported, most prominently among infants [5]. With this in mind, it is important for clinicians to familiarize themselves with evidence-based treatment regimens, supported by data from sources like the Cochrane Library. Utilizing information from numerous studies simultaneously, as in the review summarized here, supports best practice and enables physicians to effectively counsel patients.

## Abbreviations

FLG: filaggrin
IgE: immunoglobulin E
